# Dietary algal-sourced zinc nanoparticles promote growth performance, intestinal integrity, and immune response of Nile tilapia (*Oreochromis niloticus*)

**DOI:** 10.1186/s12917-024-04077-w

**Published:** 2024-06-26

**Authors:** Eman Zahran, Samia Elbahnaswy, Ahmed I. A. Mansour, Engy Risha, Ahmed Mustafa, Arwa sultan Alqahtani, Mahmoud G. El Sebaei, Fatma Ahmed

**Affiliations:** 1 Faculty of Veterinary Medicine, Department of Aquatic Animal Medicine, Mansoura University, Mansoura, 35516 Egypt; 2 National Institute of Oceanography and Fisheries (NIOF), Cairo, 11516 Egypt; 3 Faculty of Veterinary Medicine, Department of Clinical Pathology, Mansoura University, Mansoura, 35516 Egypt; 4 Department of Biological Sciences, Purdue University, Fort Wayne, Indiana 46805 USA; 5 College of Science, Department of Chemistry, Imam Mohammad Ibn Saud Islamic University (IMSIU), P.O. Box 90950 Riyadh, 11623, Saudi Arabia; 6 College of Veterinary Medicine, Department of Biomedical Sciences, King Faisal University, 31982 Al-Ahsa, Saudi Arabia; 7 Faculty of Veterinary Medicine, Department of Biochemistry and Molecular Biology, Mansoura University, Mansoura, 35516 Egypt; 8 Faculty of Science, Department of Zoology, Sohag University, Sohag, 82524 Egypt

**Keywords:** Tilapia, Nanoparticles, Feeding, Intestinal immunity, Mucin, Gene expression

## Abstract

**Background:**

Trace elements play a crucial role in fish nutrition, with zinc (Zn) being one of the most important elements. BIO-sourced zinc nanoparticles were synthesized using the green microalga *Pediastrum boryanum* (BIO-ZnNPs, 29.35 nm). 30 or 60 mg/ kg dry feed of the BIO-ZnNPs (BIO-ZnNPs_30_ and BIO-ZnNPs_60_) were mixed with the Nile tilapia (*Oreochromis niloticus*) basal diet and fed to the fish for 8 weeks to evaluate their impact on fish growth, digestion, intestinal integrity, antioxidative status, and immunity.

**Results:**

A significant enhancement was observed in all investigated parameters, except for the serum protein profile. BIO-ZnNPs at 60 mg/kg feed elevated the activities of reduced glutathione (GSH) and catalase (CAT), enzymatic antioxidants, but did not induce oxidative stress as reflected by no change in MDA level. Fish intestinal immunity was improved in a dose-dependent manner, in terms of improved morphometry and a higher count of acid mucin-producing goblet cells. **I**nterleukin-8 (*IL-8*) was upregulated in BIO-ZnNPs_30_ compared to BIO-ZnNPs_60_ and control fish groups, while no significant expressions were noted in tumor necrosis factor-alpha (*TNFα),* nuclear factor kappa B* (NF*_*k*_*B)*, and *Caspase3* genes.

**Conclusion:**

Overall, BIO-ZnNPs inclusion at 60 mg/kg feed showed the most advantage in different scenarios, compared to BIO-ZnNPs at 30 mg/kg feed. The positive effects on growth and intestinal health suggest that BIO-ZnNPs supplementation of aquafeeds has many benefits for farmed fish.

## Introduction

Essential minerals are trace elements and micronutrients crucial for animal metabolism. These microminerals intensify the metabolic processes of animals and fish, thereby improving their nutritional value [1]. However, a very narrow margin separates the essentiality and toxicity of these minerals in animal feeds [2]. The requirement for trace minerals depends mainly on the physiological status of the fish body, in addition to the mineral content of metabolic antagonistic elements (e.g., copper, iron, cadmium, molybdenum) in the host water [3].

Zinc (Zn) is one of the most important micronutrients essential for fish physiology and biology. It is one of the most abundant trace metals in fish obtained from water and/or diet through the gills and alimentary canal, and cannot be produced biologically [4]. Dietary Zn is essential for fish because it is involved in numerous metabolic pathways as a specific cofactor of several enzymatic reactions, a structural unit of non-enzymatic macromolecules, and an important component of body fluids [5]. Recently, dietary Zn has been reported to promote immuno-biochemical plasticity in fish and to provide protection against several stressors [5, 6]. Supplementation of fish feed with Zn enhances their metabolism, resulting in higher growth, survival, and production rates.

The level of Zn in freshwater and saltwater is insufficient to meet the growth requirements of aquatic species; therefore, Zn is an essential mineral and should be supplemented in fish feed to fulfill their requirements [7]. Previous studies have reported different Zn levels in different species, in carp and rainbow trout at 15-30 mg/kg [3], Atlantic salmon at 37–67 mg/kg [8], channel catfish at 20 mg/kg [9], and Nile tilapia [10]. In addition, several inorganic and organic forms of Zn are used in fish feed. The inorganic forms are usually different chemical salts containing Zn, which can have detrimental effects on both water quality and the environment and are slowly absorbed from the fish intestine. However, highly absorbed organic forms of Zn are more expensive [11, 12]. Therefore, new approaches have been adopted to use safe Zn forms with higher bioavailability and to reduce the supplemental Zn dosage whenever possible. In this context, effective nano-systems have been developed from several elemental nanoparticles (NPs), including ZnNPs, which are more effective than traditional zinc sources at lower dosages and help to indirectly prevent environmental contamination.

ZnNPs dietary supplementation has many advantages, such as promoting fish health, boosting immunity against infections [13, 14], enhancing fish growth performance and increasing survival [15]. However, chemically synthesized NPs have raised concerns about their possible toxic effects. To address this issue, dietary administration of green-synthesized NPs from bio-sources may further improve the efficacy of bioactive compounds (such as Zn) in terms of their bioavailability, delivery, and elimination [16], in addition to their effectiveness in *in vivo* applications [17]. Several studies suggest that *Pediastrum boryanum*, a green microalga of high nutritional value, maybe a suitable source of such ZnNPs due to its bioactive compounds of antioxidant and anti-inflammatory effects [18–20]. Intriguingly, the presence of metal-chelating biomolecules in algal extracts (e.g., polysaccharides, peptides, and pigments) has contributed to their successful use in biomolecular complexes for capping metal nanoparticles [21, 22]. *P. boryanum* is safe for in vivo applications, as its microalgal biomass is ranked as “Category 5,” which refers to secure or minimal toxicity [23]. Thus, it is considered a promising microalga for biotechnological, food, industrial, and pharmaceutical applications [23, 24].

*Oreochromis niloticus*, commonly referred to as Nile tilapia, is a highly favored farmed fish species in numerous countries worldwide, owing to its rapid growth rate and effortless adjustment to commercial diets [25]. Despite possessing numerous advantageous characteristics, the full production potential of Nile tilapia cannot be achieved unless its nutritional needs are fulfilled, in this case through the utilization of more bioavailable zinc forms in the aquafeed [26]. For this study, a basal diet with inorganic Zn form contained in the mineral premix was used as a reference diet. This is different from other studies that use a free-Zn mineral premix, which is scientifically controversial. Furthermore, this study was conducted as a field trial to mimic the realistic conditions in a fish farm, rather than on the experimental scale level. Considering this, we synthesized green ZnNPs using *P. boryanum* microalga for the first time, to the best of our knowledge, to clarify the effects of feeding diets containing *P. boryanum*-loaded zinc oxide nanoparticles (BIO-ZnNPs) on growth performance, digestive enzyme activity, antioxidant capacity, immune-relevant gene expression, and intestinal integrity compared to fish given a basal diet containing inorganic Zn.

## Material and methods

### Green synthesis of BIO-ZnNPs

#### Algal extract preparation

An extract of the selected green microalga, *P. boryanum*, was obtained and processed with some modifications to the previously published method of Dent et al. [27]. In the extraction unit, 100 g of algal powder was pressed into 1 L of distilled magnetized water for 2 h at 70°C. The product was then transferred to the nano-synthesis unit for the eco-friendly precipitation of BIO-ZnNPs.

#### BIO-ZnNPs precipitation

BIO-ZnNPs were precipitated using an eco-friendly synthesis method previously described by Devasenan et al. [28]. Briefly, Zn^2+^ solution (1 mM) was added dropwise to an equal volume of algal extract suspension under continuous magnetic stirring at room temperature for 2 h. The resulting precipitate was reduced under UV irradiation from a factor lamp (Vilber Lourmat-6. LC, France, λ = 254 nm) for 20 min. The reduced NPs were then filtered using Whatman no. 1 filter paper (Whatman International Ltd., Kent, UK) and stored at -18°C until use [29, 30].

#### Characterization of BIO-ZnNPs

The size and morphology of the green-synthesized BIO-ZnNPs were evaluated using Transmission Electron Microscopy (TEM) (JEOL TEM-2100, Tokyo, Japan, under an operating voltage of 200 kV) and zeta potential to illustrate their morphological characteristics, at the Electron Microscope Unit, Mansoura University, Egypt. Imaging was conducted after the solvent had evaporated, using a connected CCD camera [30]. The samples were subjected to crystallographic analysis via powder X-ray diffraction (XRD). Scanning mode X-ray diffraction patterns were captured using a Bruker D2 phaser analytical instrument set at 30 kV and 10 mA current with Cu K radiation (λ = 1.54060 Ω). Intensities ranging from 5° to 79.93° were measured at two angles. A comparison was made between the diffraction intensities and the standard JCPDS files. Furthermore, the surface charge and stability of the prepared ZnNPs were characterized using a Zetasizer Nano ZS90 Size Analyzer (Malvern Panalytical, MA, USA). Fourier-transform infrared (FTIR) spectroscopy was used to identify the functional biomolecules present in the algal extract for the reduction of Zn ions using the potassium bromide (KBr) pellet method [31, 32]. The FTIR spectra of the ZnNP samples were measured in the range 400–4000 cm^−1^ using an FTIR spectrophotometer (Thermo Fisher Scientific Nicolet IS10, USA).

### Experimental design

#### Fish rearing

Healthy Nile tilapia (*Oreochromis niloticus*), with an average body weight of 33-34g, were stocked in three concrete ponds with an area of 8 m^2^ filled with underground water. At the Fisheries Research and Application Unit, Bulteem Station Branch, National Institute of Oceanography and Fisheries (NIOF), Egypt, fish were sourced, and the trial was conducted as follows, three plastic hapas were placed in each concrete pond and the fish were stocked at a density of 10 fish/hapa (70 × 70 × 100 cm). Water quality was monitored and maintained as follows: water temperature of 26-28°C, dissolved oxygen 6.7 - 6.9 mg/L, and pH level 7-8.

#### Diet formulation

The BIO-ZnNPs suspension was mixed with dry fish feed ingredients. Basal diet ingredients and proximate analyses are presented in Table 1. After thoroughly combining all diet ingredients in a mixer (Philips HR7628, Finland), distilled water and sunflower oil were added to form a stiff dough, and the doses were determined according to a previous study [11]. The dough underwent thorough kneading before being shaped into pellets with a diameter of 3 mm using a meat mincer (ME605131 1600-Watt, Moulinex, Groupe SEB, France). Following a 24 h oven-drying period at 50°C, the pellets were sealed in plastic bags and stored at 4°C until use for feeding. Proximate chemical analysis of the experiment diet was performed as described previously [33].

**Table 1:** Basic ingredients and proximate analysis of the basal diet (air dry basis %)

**Ingredients (%)**	**Control**	**ZnNPs**_**30**_	**ZnNPs**_**60**_
Yellow corn	19.5	19.5	19.5
Soybean meal	20	20	20
Fish meal	20	20	20
Corn gluten	3	3	3
Gelatin	2	2	2
Sunflower oil	3.50	3.50	3.50
Wheat bran	30.16	30.16	30.16
Minerals and vitamins premix^a^	1	1	1
Salt	0.30	0.30	0.30
Vitamin C	0.12	0.12	0.12
Dicalcium phosphate	0.10	0.10	0.10
Methionine	0.32	0.32	0.32
ZnNPs (mg/kg)	0	30	60
**Proximate analysis (% dry matter basis)**			
Crude Protein*	32.04	32.04	32.04
Lipid*	7.06	7.06	7.06
Ca*	1.17	1.17	1.17
P*	0.53	0.53	0.53
DE (Digestable Energy)** (kcal/kg)	3016	3016	3016
Zn content (mg/Kg)	50 (in premix)	30	60

^a^The levels of the micro minerals &vitamins for tilapia are covered by supplementation of trace minerals & vitamins premixes as recommended by NRC (2011). Vitamins premix (IU or mg/kg diet); vit. A 5000, Vit.D3 1000, vit. E 20, vit. k3 2, vit. B1 2, vit. B2 5, vit. B6 1.5, vit. B12 0.02, Pantothenic acid 10, Folic acid 1, Biotin 0.15, Niacin 30. Mineral mixture (mg/kg diet); Fe 40, Mn 80, Cu 4, Zn 50, I 0.5, Co 0.2 & Se 0.2. *Analysed. ** DE calculated according to Jobling [34]The Gross energy calculated according to NRC (2011), as follow: CP×5.64+EE×9.44+NFE×4.11; whereas [Nitrogen free extract (NFE) = [100-(CP+ EE+ CF+ Ash)]. The DE was calculated according to Jobling, (1983), as follows: Digestible energy= gross energy X 0.75

#### Fish Grouping and the feeding trial

Before initiating the feeding trial and upon sampling, the fish were checked to ensure a pathogen-free status. Random samples of blood, feces, and organs (brain, kidneys, liver, spleen) were spread on blood sheep agar plates (Sigma-Aldrich, Egypt), incubated at 20°C and checked for 10 days for bacterial growth [35]. To evaluate the effects of BIO-ZnNPs-supplemented diets (Zn-free mineral premix, manually prepared for experimental diets) versus the control reference diet (Zn-sourced mineral premix, containing inorganic Zn as ZnSO_4_). A total number of 90 healthy fish in nine randomly allocated haps were assigned to the three experimental (triplicate) groups, as follows: 1) control fish group (mineral Zn form in the mineral premix, ZnSO_4_ at 50 mg/kg diet), 2) BIO-ZnNPs_30_ fish group (30 mg/kg diet), and 3) BIO-ZnNPs_60_ fish group (60 mg/kg diet). The feeding trial lasted 8 weeks. Fish were fed twice daily (at 09.00 h and 15.00 h) at 3% of their biomass (on a dry matter basis).

### Tissue sampling

Nine fish were sampled individually. Three fish were randomly caught from each replicate hapa in each group (i.e., nine fish/group). The sampled fish were euthanized using buffered MS-222 (Tricaine methanesulfonate, Finquel^®^, Argent) at 200 mg/L. Immediately, blood was withdrawn in non-heparinized tubes from the caudal vein, left for 20 min at room temperature, centrifuged at 1700 × g for 10 min (for serum separation), and serum stored at -20°C. The fish were promptly dissected, and the anterior kidney, intestine, and muscle were removed. Muscle was kept for Zn content analysis. Two sets of intestinal samples were collected. The 1^st^ was fixed in 10% buffered formalin for histopathological analysis. The 2^nd^ was homogenized in phosphate-buffered saline (PBS), pH 7.4, at 4°C and the supernatant obtained after centrifugation at 1700 × g for 15 min at 4°C was aliquoted and stored at − 80°C for subsequent digestive enzyme and oxidant/antioxidant analysis. About ~50-100 mg of the anterior kidney was preserved in RNAlater^®^ (Invitrogen, USA) solution and stored at − 80°C until gene transcriptome analysis.

### Biological analyses and measurements

#### Fish growth performance

The fish were bulked and weighed at the beginning and completion of the experimental trial to adjust the feed quantity given to the fish. Upon sampling, each fish was individually weighed and measured to determine the growth indices listed below.

Body weight gain (BWG) = mean final weight (FW, g)-mean initial weight (IW, g).

Specific growth rate (SGR, %/day) = 100 × [(Ln (mean final body weight)-Ln (mean initial body weight)]/culture period (days).

Condition factor according to the following formulae: Condition factor (K) = (W/L^**3**^) × 100; where: W = weight of fish in grams and L = total length of fish in "cm.”

#### Zinc content in fish feed and muscles

The Zn content in the fish feed and muscle was assessed according to AOAC [36]. Samples were taken at random and dried for 48 h at 105°C. The samples were then digested with concentrated H_2_SO_4_. Zinc concentration in fish feed and muscle was determined using an atomic absorption spectrophotometer (PG990, UK) using the standard method described elsewhere [37].

#### Intestinal digestive enzymes and oxidant/antioxidant activities

The intestinal homogenate supernatant was used to determine amylase activity (Bio-Diagnostics, Egypt) and lipase enzyme activity (Biorex Diagnostics, Antrim Co., Antrim, United Kingdom) according to the manufacturer’s instructions. For oxidative/antioxidant assays, malondialdehyde (MDA) levels were measured spectrophotometrically at 534 nm (Photometer 5010, Photometer, BM Co. Germany) and expressed as nmol/g. Catalase (CAT) activity was determined by measuring the decrease in hydrogen peroxide concentration at 240 nm, according to Aebi [38]. Reduced glutathione (GSH) was determined at 405 nm following Beutler [39], using Elmanns reagent (DTNB) .

#### Serum biochemical and immune parameters

Serum total protein (TP) and albumin levels were measured according to the manufacturer’s instructions with Cobas pack reagents using a COBAS INTEGRA^®^ 400 plus analyzer (Roche Diagnostics, Indianapolis, IN, USA). Serum IgM was estimated by an immunoturbidimetric assay following the manufacturer’s instruction with Cobas pack reagents using a COBAS INTEGRA^®^ 400 plus analyzer (Roche Diagnostics).

#### The expression of immune-related genes

Total RNA was manually extracted from 100 mg of the anterior kidney using a handheld homogenizer to disperse the tissue immersed in one mL of Genzol^TM^ (Geneaid Biotech Ltd, Taiwan) without DNase treatment. The pellet was dissolved in TE buffer (pH 8.0) as described previously [40]. The RNA quantity was estimated using a Nanodrop spectrophotometer (Q5000/Quawell, Massachusetts, USA). Complementary DNA (cDNA) containing 1 μg of total RNA was synthesized using the TOPscript™ RT DryMIX(dT18) cDNA Synthesis Kit (Enzynomics Co Ltd., Daejeon, Republic of Korea) according to the manufacturer's protocol. Specific primers were used to amplify selected immune-related genes of Nile tilapia according to previous studies, namely the pro-inflammatory cytokines tumor necrosis factor-alpha (*TNF-α*) and interleukin 8 (*IL-8*) [41], as well as *caspase3* [42] and nuclear factor kappa B (*NFκB*) [43], with β-actin used as the housekeeping gene. The QuantStudio™ 1 Real-Time PCR System (Applied Biosystems™ Thermo Fisher Scientific, USA) was used to quantify gene expression using Solg™ 2X Real-Time PCR Smart mix (Including SYBR^®^ Green) (SolGent Co., Ltd. Yuseong-gu, Daejeon, Korea). The thermocycling conditions were as follows: 95°C for 20 s, followed by 40 cycles of denaturation at 60°C for 40 s, and elongation at 72°C for 30 s.

#### Intestinal integrity and biometry

##### Intestinal morphometry

Intestinal tissue samples were fixed in 10% neutral buffered formalin for 24 h, embedded in paraffin wax, and sectioned at 5 µm. Selected slides were routinely stained with Hematoxylin and Eosin (H&E), according to Suvarna et al. [44] for morphometry and integrity investigations. The stained slides were examined under a light microscope (Olympus CX 31) and images were captured using a connected camera (Olympus DP 21 digital camera) (Olympus Corporation, Tokyo, Japan) for histomorphometric measurements. Intestinal morphometry, including wall thickness (crypt depth/CD), villus height (VH), width (VW), and area (VSA) was analyzed using image analysis software (Sigma Scan Pro5, SPSS INC) as described previously by Islam et al. [41]. The five highest villi per section were detected and selected for measurement. Villus height per section and tip-to-bottom length of each villus were measured. Average measurements were expressed as the mean villus height per section [45].

##### Histochemical Differentiation of the Intestinal Goblet Cells (GCs)

Semi-quantification of the different types of intestinal mucin-producing GCs was conducted via color differentiation using Alcian Blue & Periodic-Acid Schiff (AB & PAS) double staining according to Padra et al. [46] and Ahmed et al. [47] with minor modifications. In brief, some slides of the intestinal tissue were double stained with AB (pH 2.5), which stains the acid mucins blue, and PAS, which stains neutral mucins pink. GC counting was performed in triplicate sections (of every five successive sections) per treated group, along a 5000 µm length of the mucosal epithelium in triplicate fields (40 ×) per section [48]. GCs differentially stained in blue, pink, or purple (producing mixed acid/neutral mucin) and that were negatively stained (free of mucin) were counted individually under a microscope. To exclude biased evaluations, cell counting was assessed as a blinded field of well-defined goblet-like cells. The obtained data were expressed as the mean ± SD.

### Statistical analysis

Data were first subjected to normality and homogeneity checks using Kolmogorov-Smirnov and Levene’s tests, respectively. The significance between the variables of the groups was analyzed by one-way analysis of variance (ANOVA) using GraphPad Prism^®^ statistics package version 8.4.2 (GraphPad Software, Inc., USA). Normalized individual fold-change values were anchored to the lowest value recorded in each data set, and then Log2 transformed, as described previously [49]. Differences were considered statistically significant at *P* < 0.05. All data were expressed as mean ± standard error (SE) of the mean.

## Results

### Characteristics of the synthesized BIO-ZnNPs

**Fig. 1:**
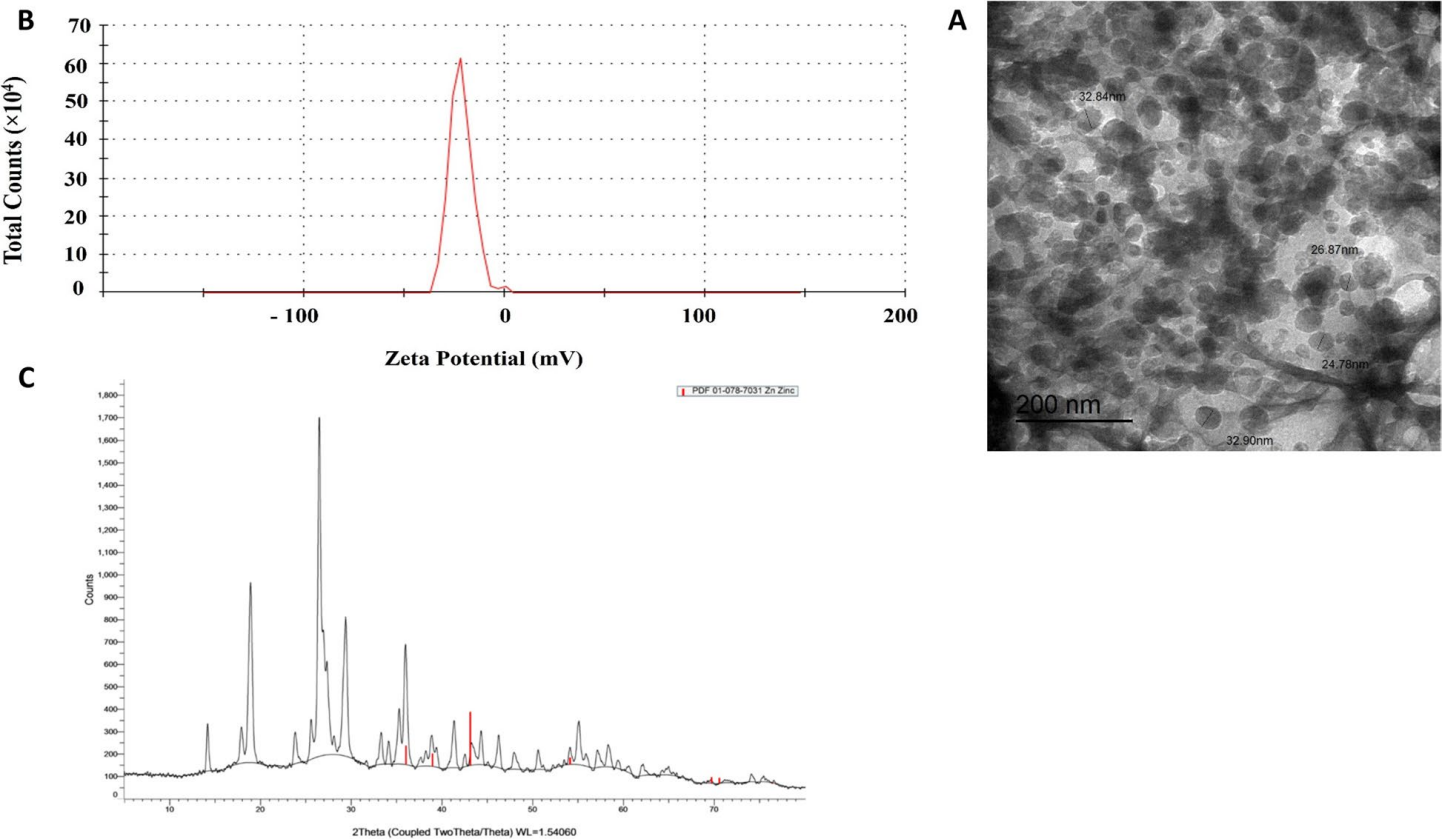
**A** TEM micrograph showing the shape and size distributions for BIO-ZnNPs. The scanned particles have a mean size of 29.35 nm. **B** Zeta potential spectrum of the synthesized BIO-ZnNPs recording -21.5 ± 5.68 mV. **C** X-ray diffraction (XRD) pattern of BIO-ZnNPs

TEM micrographs showed spherical particles with few aggregates. The mean size of the estimated particles was 29.35 nm (Fig. 1^A^). The zeta potential spectrum of the synthesized BIO-ZnNPs indicated negative charging of the particles (-21.5 ± 5.68 mV) (Fig. 1^B^). X-ray diffraction (XRD) analysis of zinc oxide nanoparticles (ZnONPs) showed a series of peaks that corresponded to the different planes of atoms in the crystal structure of ZnO. The XRD pattern showed several peaks that can be indexed to the wurtzite phase of ZnO. The peaks at approximately 31°, 34°, and 62° were the most intense. These peaks corresponded to the (100), (002), and (110) planes of the wurtzite structure of ZnO. The fact that these peaks are the most intense indicates that the ZnO nanoparticles in the sample are predominantly oriented, with their (100), (002), and (110) planes parallel to the surface of the sample (Fig. 1^C^).

### Fish growth indices

The growth performance of fish fed the BIO-ZnNPs-supplemented diets, particularly at the highest dose (60 mg/kg), was higher than that of the control group (Table 2). Consequently, FW, BWG, and SGR showed a significant increase in the BIO-ZnNPs_60_ group compared with the control fish group. The length and K factor, however, showed no statistically significant changes (Table 2).

**Table 2:** The growth performance parameters of Nile tilapia fed on 30 or 60 mg BIO-ZnNPs/kg feed or basal diets for 8 weeks

**Groups**	**Growth Indices**					
	**IBW**	**FBW**	**BWG**	**SGR**	**Length**	**K factor**
**Control**	33.33 ± 0.88	50.33 ± 0.88^b^	17.00 ± 0.00^b^	0.75 ± 0.01^b^	15.33 ± 0.33	1.47 ± 0.03
**BIO-ZnNPs**_**30**_	33.33 ± 0.33	55.00 ± 2.89^ab^	21.67 ± 2.91^ab^	0.89 ± 0.09^ab^	15.33 ± 0.33	1.53 ± 0.07
**BIO-ZnNPs**_**60**_	34.33 ± 1.45	60.00 ± 2.89^a^	26.00 ± 1.53^a^	1.01 ± 0.02^a^	16.33 ± 0.33	1.43 ± 0.03

The fish were fed the control diet (Control) or diets containing 30 mg BIO-ZnNPs (BIO-ZnNPs_30_) or 60 mg BIO-ZnNPs (BIO-ZnNPs_60_) /kg feed for 8 weeks. All data are expressed as mean ± SEM (*n* = 6/group). Different letters on the mean values in a column indicate statistically different data (*p* < 0.05) or insignificance. (IBW) initial body weight, (FBW) final body weight, (BWG) body weight gain = FBW-IBW, (SGR) specific growth rate= =100*(LN(FBW)-LN(IBW))/56, and (K-factor) condition factor = [(fish weight) (g)/ (total fish length (cm))3] × 100

### Zinc content in fish feed and muscles

**Fig. 2:**
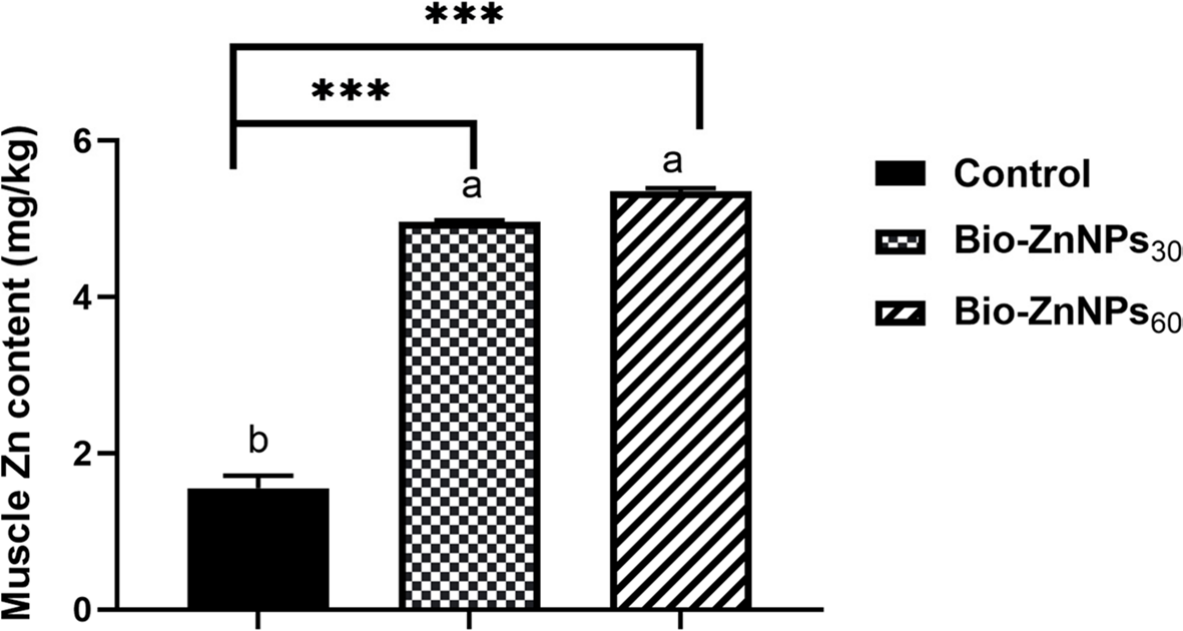
Zinc content in muscle tissue of Nile tilapia fed on 30 or 60 mg BIO-ZnNPs/kg feed or basal diets for 8 weeks. Data are presented as Mean ± SEM. Values with a different letter are significantly different between groups (ANOVA with post hoc Tukey test). Asterisks indicate the significant level *P (<0.05), **P (<0.01). ***P (<0.001)

The actual Zn content in the three diets was determined to be 55 mg/kg (ZnSO_4_, a commercial diet used as a reference feed), 35 mg/kg (BIO-ZnNPs_30_), and 67 mg/kg (BIO-ZnNPs_60_). These values were marginally higher than the added concentrations, owing to the presence of trace amounts of Zn in these ingredients. The accumulation of zinc in fish muscle was significantly enhanced in diets supplemented with both BIO-ZnNPs (*P*< 0.001) compared to fish fed the control diet. No statistically significant variation was seen between fish fed the two doses of BIO-ZnNPs (Fig. 2).

### Intestinal digestive enzymes and oxidant/ antioxidant activities

**Fig. 3:**
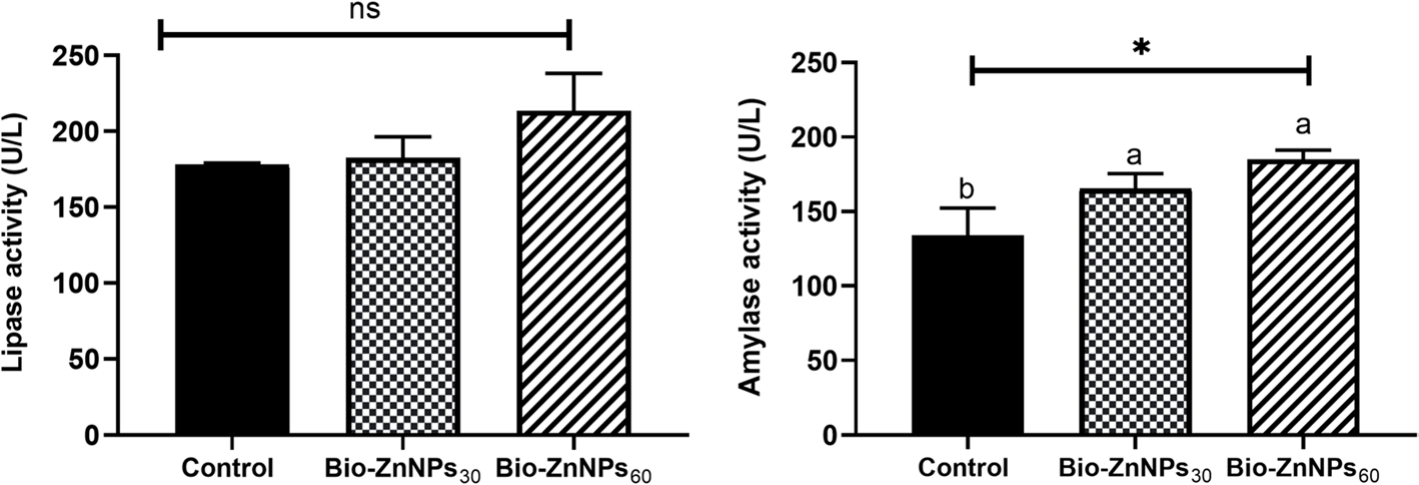
Estimated levels of the digestive enzymes’ activity observed in the intestinal homogenate of the O. niloticus fed on 30 or 60 mg/kg BIO-ZnNPs compared to non-supplemented fish for 8 weeks. Data were represented as Mean ± SEM. Values with a different letter superscript are significantly different between groups (ANOVA with post hoc Tukey test, *P (<0.05), **P (<0.01). ***P (<0.001)

Analysis of the digestive enzyme activities revealed that the BIO-ZnNPs-supplemented groups had a significant increase (*P*< 0.05) in amylase activity but no change in lipase activity when compared to the control group (Fig. 3A). The activities of the intestinal oxidant/antioxidant enzymes, MDA, GSH, and CAT, are displayed in Fig. 3B. Feed supplementation with BIO-ZnNPs (30 or 60 mg/kg) significantly augmented CAT enzyme activity (*P* < 0.05) compared to the control fish. The activity of GSH was significantly (*P* <0.05) elevated in the BIO-ZnNPs_60_ group but not the lower dose (BIO-ZnNPs_30_) group compared to the control. In both supplemented groups, no statistically significant difference was observed in the activity of MDA compared to that in the control group.

### Serum biochemical and immune parameters

**Fig. 4:**
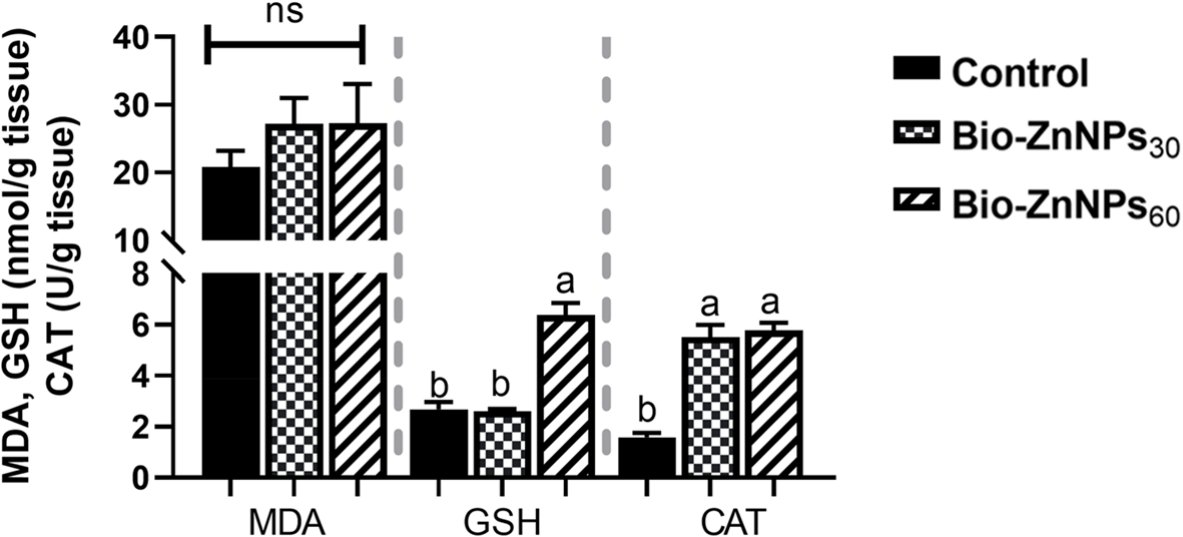
The effects of BIO-ZnNPs supplemented diets on the activity of the intestinal oxidant/ antioxidant enzymes, malondialdehyde (MDA), reduced glutathione (GSH), and Catalase (CAT), of Nile tilapia, fed diets supplemented with BIO-ZnNPs (30 or 60 mg/kg) or non-supplemented diets for 8 weeks. Data were represented as Mean ± SEM. Values with a different letter superscript are significantly different between groups (ANOVA with post hoc Tukey test, *P (<0.05), **P (<0.01). ***P (<0.001)

Profiling of serum proteins (Fig. 4) showed no significant changes (*P* > 0.05) in the levels of total protein, **a**lbumin, and **g**lobulin in the BIO-ZnNPs (30 and 60 mg/kg)-supplemented groups compared with the control fish. However, IgM levels were increased significantly (*P* < 0.01) in the BIO-ZnNPs_60_ group compared to the BIO-ZnNPs_30_ and control groups, with no statistical difference (*P* > 0.05) between the latter.

### Gene expression analysis

**Fig. 5:**
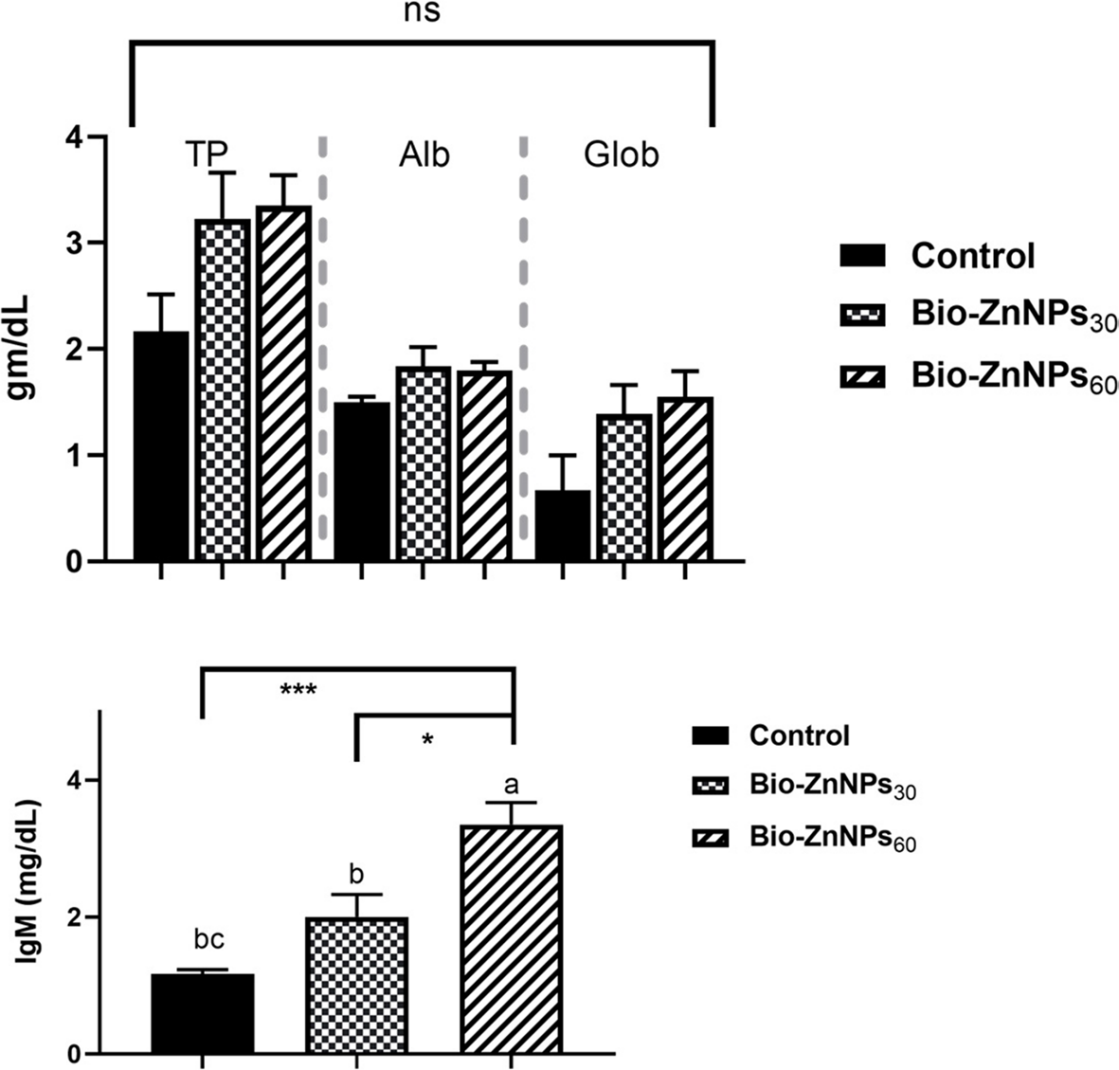
Total protein (TP), albumin (Alb), globulin (Glob), and immunoglobulin (IgM) levels in the O. niloticus fed diets supplemented with BIO-ZnNPs (30 or 60 mg/kg) or non-supplemented diets for 8 weeks. Data were represented as Mean ± SEM. Values with a different letter superscript are significantly different between groups (ANOVA with post hoc Tukey test, *P (<0.05), **P (<0.01). ***P (<0.001)

The expression levels of the immune-related genes analyzed are shown in Fig. 5. The mRNA levels of the *NF*_*k*_*B*, *TNFα*, and *Caspase3* genes showed no significant differences between the groups. However, the mRNA expression level of *IL-8* was upregulated (*P*> 0.05) in fish fed 30 mg/kg BIO-ZnNPs compared with BIO-ZnNPs_60_ and control fish groups, without statistical differences between the latter.

### Intestinal integrity

#### Intestinal histomorphometry

**Fig. 6:**
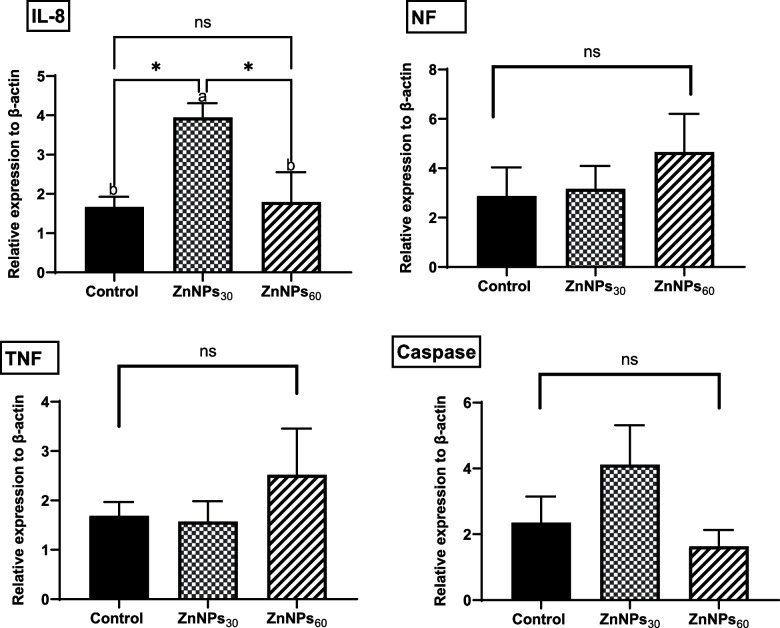
The mRNA expression levels of IL-8, NFkB, TNFα, and Caspase relative to β-actin housekeeping gene in O. niloticus fed on non-supplemented or fed with BIO-ZnNPs-30 mg/kg, or BIO-ZnNPs-60 mg/kg supplemented diets for 8 eeks. Data were represented as Mean ± SEM. Values with a different letter superscript are significantly different between groups (ANOVA with post hoc Tukey test, *P (<0.05), **P (<0.01). ***P (<0.001)

Intestinal histomorphometry of Nile tilapia revealed normal intestinal architecture (Fig. 6^A^). Dietary supplementation with BIO-ZnNPs significantly increased VSA, VH, and VH/CD compared with the control inorganic Zn-fed fish. Fish-fed BIO-ZnNPs_60_ exhibited the highest values for all measurements (Fig. 6^B^).

#### Goblet Cells (GCs) count

**Fig. 7:**
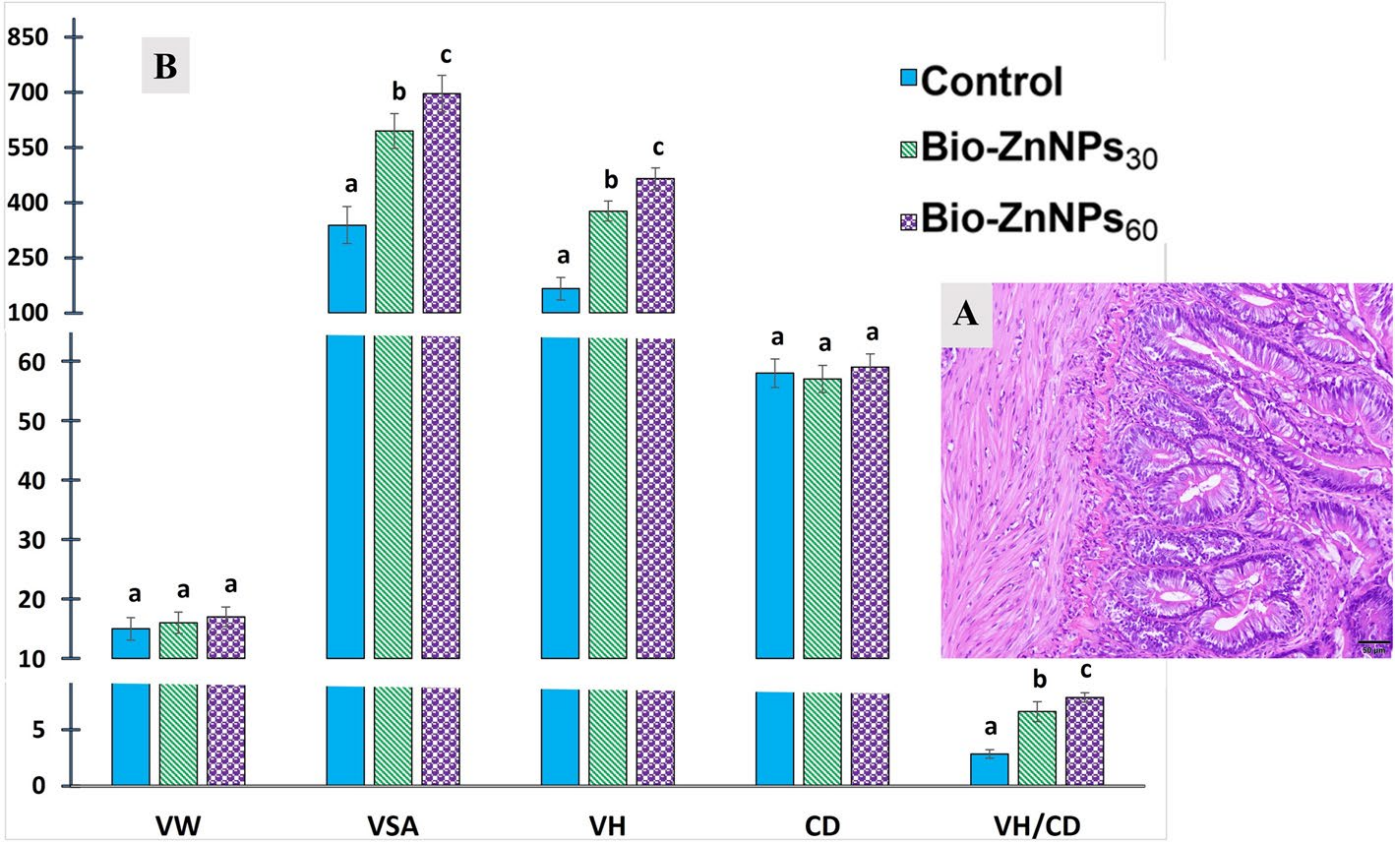
The intestinal histomorphometry of the intestine of non-supplemented O. niloticus or fed with BIO-ZnNPs-30 mg/kg, or BIO-ZnNPs-60 mg/kg supplemented diets for 8 weeks. **A** showed normal architecture of the proximal intestine. **B** Column histogram displaying the statistical analysis of the intestinal morphometric indices. Data were represented as Mean ± SEM. Values with a different letter superscript are significantly different between groups (ANOVA with post hoc Tukey test, *P (<0.05), **P (<0.01). ***P (<0.001)

**Fig. 8:**
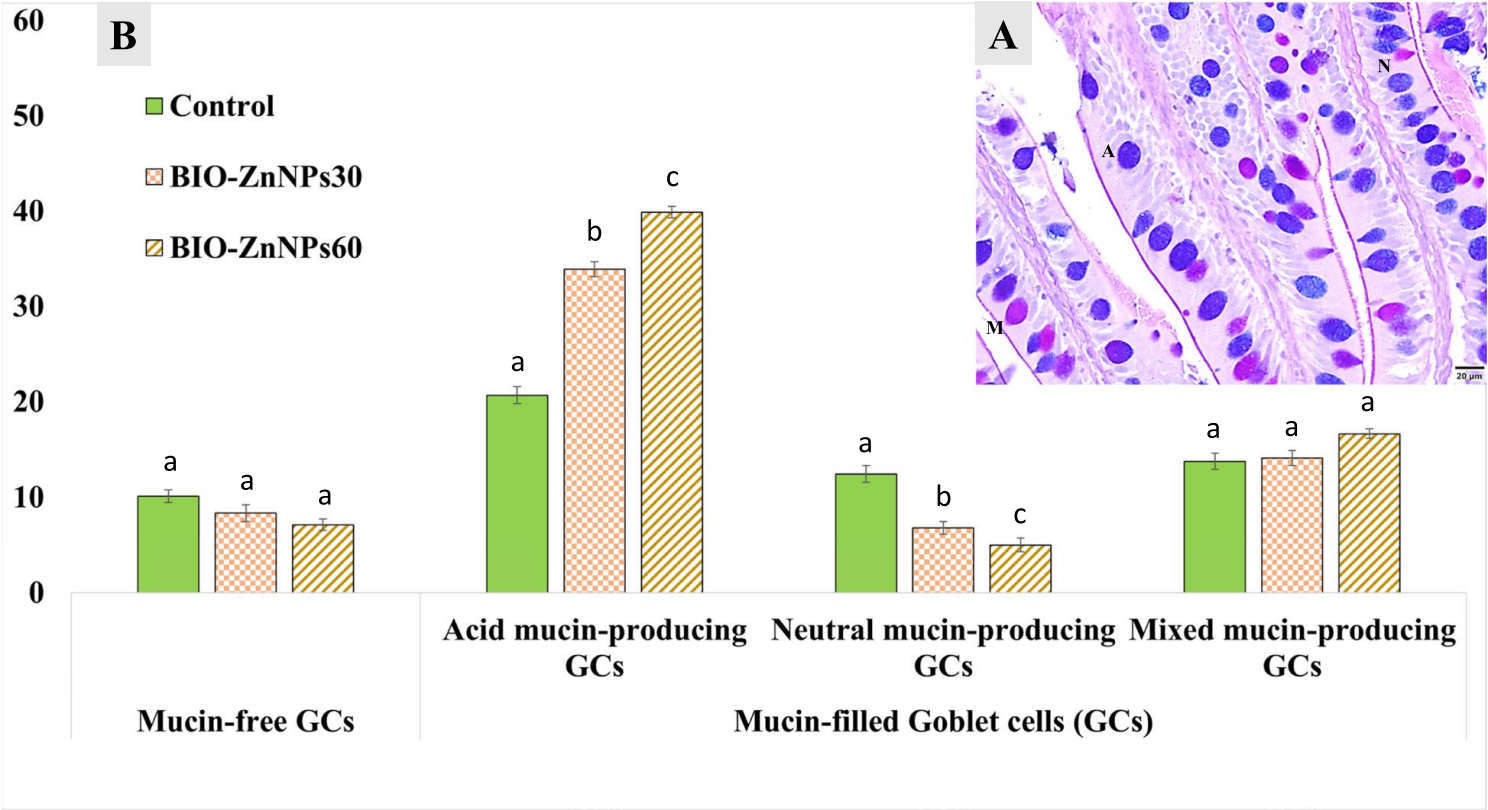
Differential count of the goblet cells (GCs) in the intestine of non-supplemented O. niloticus or fed with BIO-ZnNPs-30 mg/kg, or BIO-ZnNPs-60 mg/kg supplemented diets for 8 eeks. **A** AB & PAS double staining showing color differentiation of four types of the GCs, including mucin-free (negative stain), acid mucin-producing (blue), neutral mucin-producing (pink), and mixed mucin-producing cells (purple). **B** Column histogram displaying the statistical analysis of the GCs count. Data were represented as Mean ± SEM. Values with a different letter superscript are significantly different between groups (ANOVA with post hoc Tukey test, *P (<0.05), **P (<0.01). ***P (<0.001)

AB and PAS double staining elicited color differentiation of the four types of GCs in the fish intestine: mucin-free (negative stain), acid mucin-producing (blue), neutral mucin-producing (pink), and mixed mucin-producing cells (purple) (Fig. 7^A^). The number of mucin-free GCs showed no significant difference in the counts among control, BIO-ZnNPs_30_ and BIO-ZnNPs_60_ (10.11± 0.67, 8.33± 0.87, and 7.11± 0.6); respectively (Fig. 7^B^). However, the mucin-producing GCs showed a significant increase (*P* < 0.05) in the acid mucin-producing GCs observed in BIO-ZnNPs_30_ and (33.89± 0.78, 39.89± 0.6); respectively as compared with the control diet (20.67± 0.87), with a statistical change (*P* < 0.05) between the former. In contrast, neutral mucin-producing GCs decreased significantly (*P* < 0.05) in the intestine of fish fed the BIO-ZnNPs diets, also in a dose-dependent fashion, where BIO-ZnNPs_60_ fish group had the lowest counts (5± 0.71), followed by BIO-ZnNPs_30_ fish group (6.78± 67), compared to the control (12.44± 0.88). Lastly, regarding the mixed mucin-producing GCs, no significant effect (*P* > 0.05) was noticed in BIO-ZnNPs_30_ (14.11± 0.78) and BIO-ZnNPs_60_ (16.67± 0.5) compared to the control (13.78± 0.83) (Fig. 8^B^).

## Discussion

Implementing new eco-friendly strategies to promote nutrition in aquaculture is imperative to meet the demand for high-quality fish protein as aquaculture intensifies [50]. With their vast array of biological applications, including aquaculture, green-synthesized nanoparticles are providing innovative, well-balanced diets for fish, ensuring their optimal growth and health [51]. Additionally, green NPs are more bioavailable than other forms like chemical ones, and using ZnNPs mediated by *Pediastrum boryanum* can favor the growth and immune response of fish owing to the Zn action on the physiological body functions and also the microalga with its bioactive components [13, 20].

In the present study, the synthesized BIO-ZnNPs were spherical particles of 29.35 nm mean size that formed a few aggregates. This is similar to earlier studies where the biosynthesized Zn NPs had a mean size of less than 100 nm with few agglomerates and zeta potential in the range of +100 ​ to −100 mV [52]. XRD indicated the presence of Zn in the samples, which conformed with previous reports, where ZnONPs were green-synthesized using flower extract of *Nyctanthes arbortristis*, extracts of *Calotropis gigantea*, *Laurus nobilis,* and *Leucas aspera and* oxy-cyclodextrin *complex* [53–56].

Feeding with the BIO-Zn NPs supplemented diets significantly increased Zn bioavailability in fish muscle. Similar to these findings, Zn content has been observed in the muscle of Nile tilapia-fed biogenic ZnONPs after 12 weeks [26] and 75 days [15]. In contrast, Shahpar and Johari [57] reported that the total zinc content of rainbow trout larvae was highest when they were fed mineral ZnSO_4_ as opposed to ZnONPs and organic Zn. Dekani et al. [58] found that the liver possessed the highest concentration of Zn across all forms, whereas the concentration was lowest in the muscles. This result indicates that BIO-ZnNPs can traverse cellular and nuclear membranes because of their small size, substantial surface area, and enhanced zinc accessibility at the nanoscale [26]. Indeed, based on previous research and the findings of the current investigation, it is possible to deduce that the chemical form of zinc influences its bioavailability in the body. However, to compare this study with previous studies, the digestive capacities of fish at various life stages must be considered. Furthermore, the detected Zn residues in Nile tilapia muscle recorded in our study matched the safe range for Zn content in freshwater fish muscle [59, 60], suggesting no hazard to human consumption.

In terms of the impact on growth, fish-fed BIO-ZnNPs, especially at 60 mg/kg, exhibited higher growth performance (i.e. enhanced FW, BWG, and SGR), demonstrating the potential of nanotechnology to enhance the health and production of fish. In agreement with our study, 60 mg/kg ZnONPs gave the highest specific growth rates (4 fold above the control) in Nile tilapia fed the supplemented diet for 120 days [61]. Similarly, 60 days of feeding on a diet supplemented with 10 – 50 mg/kg of Zn NPs improved the nutrient metabolism and growth performance of Rohu, and *Labeo rohita* [62]. Additionally, Nano-ZnO dietary supplementation at 20 mg/kg positively influenced the health of rohu relative to fish-fed ZnSO_4_ [63]. Faiz et al. [64] also reported higher growth performance in juvenile grass carp fed a Nano-ZnO-supplemented diet compared to those fed inorganic Zn. There is a concomitant relationship between digestive enzyme enhancement and growth performance. The former plays a crucial role in numerous physiological processes and metabolic activities in the body, including nutrient absorption and utilization, which may account for our findings. Digestive enzyme activity in this study showed that amylase activity was significantly enhanced by the BIO-ZnNPs supplementation, but that lipase activity was unchanged. Previous studies have shown improvement in amylase and lipase activities upon feeding Nano-ZnO in tilapia at 60 mg/kg [11] and 30 mg/kg [26], and in rohu-fed ZnONPs at 10 mg/kg [50]. It is not clear why lipase was unaffected in this study, but perhaps the timing of sampling or dose given was not optimal. However not assessed herein, the ability of zinc to inhibit pathogenic microbiota and promote beneficial species is significant since it facilitates digestion and nutrient absorption [65].

The BIO-Zn NPs used in this study appeared to be safe concerning the serum protein profile of Nile tilapia, as reflected by the unchanged total protein, albumin, and globulin levels compared to the control fish. These results support earlier findings of zinc oxide nanoparticles synthesized by *Nelumbo nucifera* and given to Nile tilapia [66] and ZnONPs given to broilers and weaned piglets [67, 68]. However, IgM levels increased significantly in a dose-dependent manner, suggesting higher immunity in these fish [47]. Similar findings were reported for Nile tilapia fed a diet supplemented with 30 or 60 mg/kg ZnONPs for 120 days [61] or 30 mg/kg ZnONPs for 60 days. These findings indicate that Zn acts as an essential factor that enhances humoral immunity potentially via Zn-dependent transcription factors [61, 69, 70].

As observed, dietary BIO-ZnNPs positively influenced the antioxidant enzyme activity in the intestine, where the activity of the enzymatic antioxidant GSH was increased in the BIO-ZnNPs_60_ fish group, while CAT activity increased in both supplemented groups. These findings indicate a better antioxidative response in fish fed the BIO-ZnNPs, especially the higher dose. Higher CAT activity indicates a higher rate of catabolic activity and detoxification induced by BIO-ZnNPs. CAT and GSH are the prime antioxidative enzymes in animal cells, acting to detoxify reactive oxygen species (ROS) and catalyze toxic H_2_O_2_ to biologically safe H_2_O and O_2_ [71]. Ibrahim et al. [66] reported similar results after feeding the same levels of ZnONPs synthesized from *Nelumbo nucifera* to Nile tilapia for 84 days, and in tilapia-fed ZnONPs at 30 mg/kg for 12 weeks, where a significant increase in CAT and GPx was reported [26]. Increased activities of CAT, GST, and GPx were also evident in *Pangasianodon hypophthalmus* fed ZnNPs synthesized from fishery waste [5] and in beluga (*Huso huso*) fed chitosan-ZnONPs for 28 days Gharaei et al. [72]. However, no significant difference in the activity of the MDA enzyme was found in the present study in either of the supplemented groups, which is consistent with the findings of Gharaei et al. [72], suggesting that dietary BIO-ZnNPs did not induce oxidative stress. These findings highlight the role of zinc ions as ROS-reducing agents, structurally involved in antioxidants, incorporated into thiol group proteins, and modifying the induction of metallothionein [11, 16, 26]. Indeed *P. boryanum* extracts- mediated NPs synthesis in this study, have the highest radical scavenging activity owing to their phenolic compounds, which function as natural antioxidants by counteracting reactive species of nitrogen and oxygen, thereby preventing lipid oxidative damage [19, 20].

Fish immunomodulation by feed supplementation with BIO-ZnNPs was evaluated by analysis of the expression of inflammatory-relevant genes (*IL-8*, *NF*_*k*_*B*, and *TNFα)* and an apoptotic-relevant gene (*Caspase3*). The mRNA expression level of *IL-8* was higher in the BIO-ZnNPs_30_ fish group compared to the BIO-ZnNPs_60_ and control fish groups. However, no differences in expression level were found for the *NF*_*k*_*B*, *TNFα*, and *Caspase3* genes across all fish groups. *IL-8* is a pro-inflammatory cytokine that plays a key role in the immune response and inflammation [73]. The upregulation of *IL-8* indicates that BIO-ZnNPs might exert an immunomodulatory effect, possibly participating in the regulation of fish immune responses. Our findings are consistent with other studies showing that dietary ZnONPs at the same dosages can upregulate the expression of the *IL-8* gene in Nile tilapia [74]. *TNFα* is a potent pro-inflammatory cytokine that contributes to the inflammatory response [75], whilst *NFκB* is a key regulator of cytokine expression and is closely associated with ROS generation and apoptosis [76]. In consistence with our study, no alteration has also been found in splenic mRNA expression levels of *NFkB, TNFα* [77]**,** and *caspase-3* [78] in catfish supplemented by ZnNPs at 30 mg/kg. Dietary ZnNPs can inhibit the *NFκB* signaling pathway, reduce immune cell differentiation, and suppress inflammatory mediators (such as *TNF-α* and *Caspase3*) [79, 80]. Therefore, the lack of alterations in *NF*_*k*_*B*, *TNFα*, and *Caspase3* expression in the present study may reflect the absence of oxidative stress, as indicated by the unchanged MDA levels, and evidenced histologically (see below).

Diverse alterations in the morphometry and histopathology of fish tissues subjected to various regimens and quantities of feed additives have been reported [81, 82]. Indeed, the use of feed additives can impact the intestinal tissue, with the potential to improve fish health and enhance immune status. In the present study, no histopathological alterations were observed in the BIO-ZnNP-fed fish group. However, the intestines of Nile tilapia supplemented with BIO-ZnNPs showed increased values of VH, VH/CD, and VSA compared to the control fish group, suggesting enhanced nutrient absorption with subsequent growth improvement as evidenced herein. These findings can be attributed to the small size and large surface area of ZnONPs, which promote the absorption and digestibility of nutrients in the intestine, giving an improvement in intestinal health and integrity. The improvement of intestinal health, as assessed by morphometry, has been seen using a variety of metallic NPs as diet supplements [51, 65, 83], and appears to be a common benefit of such treatment.

Intestinal mucin-filled GCs play a pivotal role in the intestinal innate gut immune system [84], reflecting the fish's intestinal health status as influenced by the received feed. Acidic mucins reinforce the mucosal barrier of the intestine and protect tissues from invading pathogenic bacteria [84], and we noticed its increase upon BIO-ZnNPs supplementation in a dose-dependent manner. This finding conforms with previous studies in Nile tilapia [27] and golden pompanos [67] fed BIO-ZnONPs or Nano-ZnONPs respectively. In addition, a significant increase in the number of acid mucin-producing GCs in rainbow trout intestines was reported following dietary enrichment with chitosan nanoparticles, which mitigated their systemic inflammatory responses against disease [35, 47].

## Conclusions

To our knowledge, this is the first report to detail the application of *P. boryanum* extract in the green synthesis of ZnNPs for Nile tilapia. The BIO-ZnNPs demonstrated more bioavailability. The inclusion of BIO-ZnNPs into the nutritional regimens of Nile tilapia yielded a variety of benefits, including enhanced growth performance and improved intestinal health and integrity, as evidenced by increased levels of digestive enzymes, antioxidant status, and intestinal integrity. Notably, no negative alterations in gut morphology or induction of inflammatory mediators were seen in fish fed the BIO-ZnNPs. The higher supplemented dose of BIO-ZnNPs showed the most promising effects. The obtained results confirmed the safety of using Bio-ZnNPs as an aquafeed supplement for supporting fish growth, and immunity and boosting their production.

## Data Availability

All data supporting the findings of this study are available within the paper.
